# Community perspectives on family planning service quality among users and non-users: a qualitative study from two cities in Burkina Faso

**DOI:** 10.1186/s12978-023-01618-8

**Published:** 2023-05-17

**Authors:** Fiacre Bazie, Ilene S. Speizer, Sarah Castle, Kindo Boukary, Amelia Maytan-Joneydi, Lisa M. Calhoun, Yentema Onadja, Georges Guiella

**Affiliations:** 1 Institut Supérieur des Sciences de la Population (ISSP), Université Joseph Ki-Zerbo, Ouagadougou, Burkina Faso; 2 Department of Maternal and Child Health, University of North Carolina at Chapel Hill, Chapel Hill, NC USA; 3 Carolina Population Center, University of North Carolina at Chapel Hill, 123 W. Franklin St., Chapel Hill, NC 27516 USA; 4 Independent Consultant (SRHR), London, UK

**Keywords:** Quality, Family planning, Burkina Faso, Qualitative, Provider interactions

## Abstract

**Background:**

Most studies that focus on quality of family planning (FP) services collect data from facilities. These studies miss the perspectives of quality from women who do not visit a facility and for whom perceived quality may be a barrier to service utilization.

**Methods:**

This qualitative study from two cities in Burkina Faso examines perceived quality of FP services from women who were recruited at the community level to avoid potential biases based on recruiting women at facilities. Twenty focus group discussions were undertaken with varying groups of women of different ages (15–19; 20–24; 25+), marital statuses (unmarried; married), and current modern contraceptive use experiences (current non-users; current users). All focus group discussions were undertaken in the local language and transcribed and translated into French for coding and analysis.

**Results:**

Women discuss FP service quality in a variety of locations, depending on their age group. Perspectives on service quality for younger women are often informed by others’ experiences whereas for the older women, they are formed by their own and others’ experiences. Two important aspects of service delivery emerge from the discussions including interactions with providers and selected system-level aspects of service provision. Important components of provider interactions relate to (a) the initial reception from the provider, (b) the quality of counseling received, (c) stigma and bias from providers, and d) privacy and confidentiality. At the health system level, discussions revolved around (a) wait time; (b) stockouts of methods; (c) costs of services/methods; (d) the expectation for tests as part of service provision; and (e) difficulties with method removal.

**Conclusions:**

To increase contraceptive use among women, it is crucial to address the components of service quality they identify as related to higher quality services. This means supporting providers to offer services in a more friendly and respectful manner. In addition, it is important to ensure that full information is provided to clients on what to expect during a visit to avoid false expectations that lead to poor perceived quality. These types of client-focused activities can improve perceptions of service quality and ideally support use of FP to meet women’s needs.

## Plain Language Summary

Quality of family planning services typically is captured from women visiting a family planning facility and thus misses the perceptions of women who may perceive quality as poor and choose not to visit a facility. This qualitative study collects focus group discussion data from women recruited at the community level to understand varying women’s perceptions of family planning service quality. Different groups were undertaken with younger women, older women, married women, unmarried women, current non-users of contraception, and current users of contraception. A total of twenty focus groups were undertaken. Findings show that women discuss FP service quality in a variety of locations, depending on their age and marital status group. Perspectives on service quality for younger women are often informed by others’ experiences whereas for the older women, they are formed by their own and others’ experiences. Women reported the importance of provider interactions as a key element of service quality. This included the provider being respectful and welcoming, the quality of counseling the provider offered, that providers did not stigmatize clients, and that providers offered services that were private and confidential. Participants also valued short wait times for services, availability of methods, low cost or free methods and services, and the ability to remove a method when they wanted. Undertaking programs that address women’s perceptions of quality can lead to improved services and ideally help programs to meet the family planning needs of all.

## Abstrait

**Contexte:**

La plupart des études axées sur la qualité des services de planification familiale (PF) se font à partir des données collectées dans les services de santé. Ces études ne tiennent pas compte des perspectives de la qualité chez les femmes qui ne sont pas utilisatrices et pour qui la qualité perçue peut constituer un obstacle à l’utilisation des services.

**Méthodologie:**

Cette étude qualitative réalisée dans deux villes du Burkina Faso examine la qualité perçue des services de PF chez les femmes recrutées au niveau communautaire afin d’éviter les biais potentiels liés à la présence dans les services de santé. Vingt focus group ont été organisés avec des femmes de différents groupes d’âge (15–19; 20–24; 25+), état matrimonial (célibataires; mariées) et expériences actuelles d’utilisation des contraceptifs modernes (non-utilisatrices actuelles; utilisatrices actuelles). Toutes les discussions de groupe ont été organisées en langue locale et transcrites et traduites en Français à des fins de codage et d’analyse.

**Résultats:**

Les femmes discutent de la qualité des services de PF dans divers endroits, en fonction de leur groupe d’âge. Les points de vue sur la qualité des services chez les femmes plus jeunes se construisent à partir des expériences des autres, tandis que pour les femmes plus âgées, ils sont construits à partir de leurs propres expériences et celles des autres. Deux aspects importants des services ressortent des discussions, notamment les interactions avec les prestataires et certains aspects des services offerts. Les éléments importants des interactions avec les prestataires ont trait (a) à la l’accueil du prestataire, (b) à la qualité du counseling, (c) à la stigmatisation et aux préjugés des prestataires, et (d) à la vie privée et à la confidentialité. Au niveau du système de santé, les discussions ont porté sur (a) le temps d’attente; (b) les ruptures de stock de méthodes; (c) les coûts des services/méthodes; (d) l’attente pour des testes sanguins dans le cadre de la prestation de services; et (e) difficultés liées au retrait de certaines méthodes.

**Conclusions:**

Pour accroître l’utilisation de contraceptifs chez les femmes, il est crucial de s’attaquer aux composantes de la qualité des services qu’elles identifient comme étant liées à des services de meilleure qualité. Cela signifie aider les prestataires à offrir des services de manière plus amicale et respectueuse. De plus, il est important de veiller à ce que les clientes reçoivent une information complète sur ce à quoi s’attendre lors d’une visite afin d’éviter les fausses attentes qui mènent à une mauvaise qualité perçue. Ces types d’activités axées sur le client peuvent améliorer la perception de la qualité des services et, idéalement, soutenir l’utilisation de la PF pour répondre aux besoins des femmes.

## Background

Previous studies have demonstrated that the quality of contraceptive services is associated with contraceptive adoption and continuation [1–8]. While quality of care is not consistently measured, researchers often use indicators or scores to assess the fundamental elements of quality proposed in 1990 by Judith Bruce [9]. These include the following components: choice of methods; information given to clients; provider technical competence; interpersonal relations; follow-up/continuity mechanisms; and appropriate constellation of services. Most of the studies examining quality of care and its relationship to family planning (FP) outcomes focus on quality as measured through facility audits, client exit interviews, provider–client observation, or mystery clients [10–13]. The approaches to measuring FP quality of care through client reports may be biased toward positive client experiences due to courtesy bias (i.e., the client has just received the service at the facility) and may not capture the potential negative experiences, for example, of disrespect and abuse (e.g., procedures provided without consent and being yelled at or scolded) [10, 14, 15]. One study from Burkina Faso among primary health care clients from urban and rural facilities demonstrates that while there is a positive response bias, the clients did report negative perspectives on health personnel practices and conduct [10]. The authors of this Burkina Faso study remark that typically the interpersonal dimensions of quality are overlooked, and more technical aspects of care are the focus for programming [10]. This may reflect that technical aspects are easier to change than interpersonal aspects; however, recently, greater attention is being paid to person-centered health care and improving the multiple dimensions of quality of care that matter to facility users [16].

As most studies focus only on clients who arrive at a facility, standard quality measures are biased toward women who are the most motivated to seek services or most in need of services. Thus, quality of care measures may be biased because they reflect users’ experiences and miss the perspectives of women and others who may choose not to visit a facility because of perceived poor quality. In addition, prior research has observed that surveys of client satisfaction and clients’ perceived quality of care likely reflect clients’ a priori expectations for the care they will receive [17]. Where initial expectations are low for quality services, clients may report high quality (or satisfaction), even if the services offered were of low quality [18].

Beyond client experiences, prior research has demonstrated that perceptions of service quality are a key driver of health care service use in domains other than family planning [19, 20]. Perceptions of health care quality are informed by interconnected community, health-system and individual factors that interact through social networks and information exchange [19]. It is important to improve quality of services, but if the target community does not perceive services to be of high quality, they may not use the services [19]. Thus, it is important to measure perceived quality of health care services at the community level to determine whether this is a barrier to actual service use.

To date, only a small number of quantitative studies examine quality of services from the community perspective to determine whether and how perceived quality is associated with reproductive health behaviors. An early study by Mroz et al. [21] from rural Tanzania measured perceived quality of services at the local health facility and found that contraceptive use was higher in communities with higher perceived quality than in communities with lower perceived quality. Similarly, a study from Tanzania demonstrated that where perceptions of service quality were higher, institutional childbirth was more common [22]. Likewise, in Malawi, among women who had a recent birth, both general perceptions of facility quality as well as actual experiences at the facility were associated with higher FP service satisfaction [23]. Finally, research in Bangladesh found that women who perceived positive health care provider skills and competence and those who reported satisfaction with provider interactions were more likely to use community clinics than their counterparts with lower scores with regard to these indices [20].

Recently, a small number of studies from Zambia have examined community perspectives of quality of services using qualitative data with a view to informing community participation in FP programming [24–26]. The authors recognize the importance of community participation as a key principle of provision of FP services within a human rights framework and thus seek to better assess person-centered perspectives on quality to support social accountability models of programming. Silumbwe et al. [25] demonstrate that health system barriers including distance to facilities, stockouts, provider bias, and appropriate infrastructure for provision of services were key factors identified by communities and providers related to perceived service quality. Thus, the authors recommend the need for additional studies from the perspective of the community to improve client satisfaction and service utilization [25]. This study from Burkina Faso attempts to fill this knowledge gap by using recent qualitative data that explore community perceptions of facility quality to expand our understanding of what community members consider higher (or lower) quality FP services. The objective of the present qualitative study is to examine how women (users and non-users) conceptualize quality of FP services and to determine whether perceptions of service quality differ between groups of women stratified by age, marital status, and FP use experience.

## Methods

### Context

This qualitative study was undertaken in Burkina Faso, a West African country with a population of about 21.5 million in 2020 [27]. Burkina Faso has seen increases in modern contraceptive use over the last 10 years, starting from 18% among married women in 2012 and increasing to 32% by early 2021 [28]. Since 2012, the government of Burkina Faso has been a key partner in the FP2020 (now FP2030) initiative and has made important commitments to increasing contraceptive use in the country. This has included adopting a policy that makes family planning (FP) free in public facilities, reducing stockouts and ensuring clinical training, equipment, and supplies, supporting community-based services, and providing financial resources to family planning programming [29].

Data were collected in November 2021 in two cities of Burkina Faso: Banfora (Cascades region) and Bobo-Dioulasso (Hauts-Bassins region). These sites were selected because they overlapped with the Beyond Bias program being led by Pathfinder International (https://www.pathfinder.org/projects/beyond-bias/). The objective of the Beyond Bias program was to reduce provider bias for provision of family planning services, particularly for young people. Briefly, the Beyond Bias intervention involved training providers to offer family planning services to all women in need, no matter their age, parity, or marital status. All intervention activities happened at the facility level with no specific outreach activities. Thus, we sought to examine perceptions of quality of care in catchment areas around facilities where providers were trained by the Beyond Bias program (i.e., where perceptions of service quality may be better) and catchment areas around facilities where providers were not trained (i.e., where perceptions of service quality may be worse). The study focused on two predominately urban areas, to limit the heterogeneity in the sample population. In this study, no differences were observed in reports of service quality by study community (Banfora versus Bobo-Dioulasso and intervention/comparison site) and thus we talk about the data jointly across the cities and intervention arms.

### Data collection

For this study, focus group discussions (FGD) and in-depth interviews (IDI) were undertaken by the Institut Supérieur des Sciences de la Population (ISSP) at the Université Joseph Ki-Zerbo in Ouagadougou in collaboration with the Carolina Population Center at the University of North Carolina at Chapel Hill in November 2021. A total of 20 FGD were carried out with women and 4 with men. In addition, 10 IDI were undertaken with adolescents (ages 15–24) and 6 with female community leaders. The focus of this paper is the information generated from the FGD with women. Each FGD comprised 8–12 participants. As shown in Table 1, groups were created to be homogenous based on age (< 25 and 25+), marital status (never and ever married), and modern family planning use experience (current non-user or current user). It is worth noting that we had more groups that included younger and unmarried sexually experienced women as we were interested in their perceptions of service quality and whether this was a barrier to their service use.

**Table 1:** Description of sample of sexually experienced women included in FGD qualitative data collection, Bobo Dioulasso and Banfora, Burkina Faso 2021

Group number	Age group	Marital status	Current use status*	Region of residence	Number of participants
1	15–19	Never married	Non-user	Banfora	9
2	15–19	Never married	Non-user	Banfora	12
3	15–19	Never married	Non-user	Bobo-Dioulasso	9
4	15–19	Never married	Non-user	Bobo-Dioulasso	10
5	15–19	Never married	Current user	Banfora	8
6	15–19	Never married	Current user	Banfora	8
7	15–19	Never married	Current user	Bobo-Dioulasso	9
8	15–19	Never married	Current user	Bobo-Dioulasso	10
9	20–24	Never married	Non-user	Banfora	8
10	20–24	Never married	Non-user	Bobo-Dioulasso	8
11	20–24	Never married	Current user	Banfora	10
12	20–24	Never married	Current user	Bobo-Dioulasso	8
13	15–24	Ever married/in union	Non-user	Banfora	9
14	15–24	Ever married/in union	Non-user	Bobo-Dioulasso	9
15	15–24	Ever married/in union	Current user	Banfora	9
16	15–24	Ever married/in union	Current user	Bobo-Dioulasso	8
17	25–49	Ever married/in union	Non-user	Banfora	12
18	25–49	Ever married/in union	Non-user	Bobo-Dioulasso	11
19	25–49	Ever married/in union	Current user	Banfora	12
20	25–49	Ever married/in union	Current user	Bobo-Dioulasso	11

*Current users of modern method includes using oral contraceptive pill, injectable, implant, and IUD. Women who only reported condom use were considered non-users since this is not a facility-based method. Some non-users had used previously but were not using at the time of the FGD

Participants were recruited at the community level (i.e., not through facility contacts) to provide a perspective of family planning services among users and non-users of contraception. Prior to data collection, the study team reached out to the six target communities (two near a Beyond Bias facility and one not near a Beyond Bias facility in each city) where the data would be collected to identify community leaders and persons involved in women’s associations. These individuals were asked to help with recruitment and to help identify appropriate locations to undertake the FGD sessions. Community leaders were told about each of the target groups (in terms of age and marital status) and asked to help mobilize a specific target group on a certain day. At the time of the meeting, about 20 women who met the age/marital status criteria were invited and interviewers screened each woman privately on her contraceptive use to determine if she would be eligible to be in a FGD for her age/marital status and use status. Because the focus of this study is on facility-based quality of services, women who reported that they had only ever used condoms were put into the “non-user” group while current users of other modern methods (IUD, implant, injectables, pills) were put into the “user” group. A recruitment script was used for this screening step and those women who were eligible for a FGD but not the one on the specific day were given an appointment for another day/time. Those who did not meet the criteria were thanked and given a refreshment.

Upon identification of a group of 8–12 participants for a specific group, the interviewers went on to obtain signed informed consent from each participant prior to moving into the FGD that included a trained moderator and a trained note taker. Prior to starting the group discussion, the moderator shared the ground rules of the session including that people would not use names but rather identification numbers (e.g., 1–12), that any information shared in the discussion should not be discussed outside the group, and that the discussions would be recorded. All FGD were recorded for subsequent translation and transcription in French. The FGD lasted about 90 min and at the end, all participants received a refreshment.

The study used a carefully pre-tested semi-structured interview guide that covered the types of family planning services available in the community, where women of different ages and marital statuses feel comfortable seeking services, what aspects of services women appreciate or do not appreciate, examples of positive and negative experiences or perspectives with the facility, and where women discuss perceptions of service quality. The FGD also provided depth on perspectives of facility quality and why some women who have a need for FP are not using FP services and if non-use of services relates to perceptions of facility quality. Coding and analysis of FGD was undertaken in French and was performed jointly by the members of the study team in Burkina Faso, the U.K., and the U.S. using Dedoose, a collaborative qualitative data analysis software. Matrices were developed to identify themes, connections, and patterns around how women conceptualized various elements of FP services and quality. Thematic analysis methods were used to examine perceptions of facility quality by the various characteristics of the groups.

All study materials including consent forms, FGD guides, and the study protocol were reviewed and approved by the ethics committee in Burkina Faso (Comite d’Ethique pour la Recherche en Sante #2021-000133) and at the University of North Carolina at Chapel Hill (#21-1898).

## Results

During the focus groups with women, perceptions of quality were identified through questions that focused on points of satisfaction, dissatisfaction, and women's expectations around FP services in their community. Below, we summarize the results in two parts. First, we discuss how women’s perceptions of quality are constructed and second, we identify the aspects of services that are most closely linked to quality perceptions.

### Construction of notions of family planning service quality among women

The results of the FGDs show that according to the socio-demographic profiles of women, perceptions of quality are constructed in a variety of ways. Older and married women tend to evoke their perceptions of quality from their own positive or negative experiences with services. They also refer to discussions they have had with friends or family about these experiences. Younger women and those who are single, typically have less direct experience with FP services and learn about what to expect from listening to conversations with older family members or more experienced friends. Typically, young women are learning about these experiences from their female networks that include their friends, family members, or other female community members.

In the FGDs, it was reported that FP topics are discussed in a variety of scenarios and in different locations where women often congregate among themselves. In community settings (e.g., among neighbors, at the market, or at school), the topic of contraception may be raised following discussions about unwanted pregnancies and their consequences on the household and health. It was reported that women's exchanges about FP generally focus on the advantages and disadvantages of contraception, including side effects, but also on experiences and interactions with providers and their impressions of facility-based services more generally.

FGD participants also revealed that among older women and those who are married, FP does not seem to be a taboo subject and they discussed it easily with family and friends. These exchanges take place at the market, in health centers or when women meet in women's spaces such as association meetings, hairdressing salons or friend groups.

*"The women's talk does not end. And there is no fixed place to talk. At times, if you cross two or three others, you know that a conversation will start. You may be discussing something else and then this is raised. And each wants to show her knowledge to the other. Or often, there are women who every time you see her, she is pregnant. In this case, if it is an easily accessible person and you can talk to her without there being any problem, you can also explain it [FP] so that this person can rest [from pregnancy]. Because motherhood is not easy. Often also in tontine places, in women's meeting places, you may be talking and you will address this topic. That's it, there is no fixed place."*

Bobo Dioulasso, married women, FP users, ages 25–49

Among younger and unmarried women who have less experience with services, exchanges about FP use and FP services take place either in the community or at school when there are discussions of an unwanted pregnancy of one of their peers or when they are looking for contraceptive methods for themselves.

*“It can happen that if you see one of your peers who is pregnant and you start talking about pregnancy, it is at this time we often enter these topics. It is from these talks that we take advantage of giving each other advice so that another among us does not get pregnant too and regret it later.”*

Banfora, unmarried women, non-users, ages 15–19

The young women also learn about contraception and FP services in the community by listening to older women discussing these topics either within the family or in community settings (e.g., the market).

*"You can learn this [about FP] from grown-up people too, you can sit in the courtyard and experienced women talk about it so you enjoy listening. Or you might be sent on an errand and while out you hear people talking about this or by sitting with the elderly in the courtyard or in the hair salons also when you go to do your hair they talk about it [FP] and you listen.”*

Banfora, unmarried women, non-users, ages 15–19

But unlike older women, young people find it difficult to discuss FP, because of the fear of social stigma of discussing it in a group. This is especially a problem for those who need contraception because they fear being perceived as having loose morals. In this case, they prefer more confidential exchanges between one or two friends.

*“Married women like talking about it in groups under the shade of trees. But us young girls, we would never do that. If I tell you one-to-one that I am using family planning, you will keep quiet! It’s a secret! In town everyone knows that women use it and that young girls use it - but the young girl who will admit to it (in a group discussion) does not exist!”*

Bobo Dioulasso, unmarried women, users, ages 20–24

### Perceptions of quality

Overall, when women shared their views and concerns about FP services, two aspects of service delivery emerge—namely, interactions with providers and selected system-level aspects of service provision. Important components of provider interactions relate to (a) the initial reception from the provider, (b) the quality of counseling received, (c) stigma and bias from providers, and d) privacy and confidentiality. At the health system level, discussions revolved around (a) wait time; (b) stockouts of methods; (c) costs of services/methods; (d) the expectation for tests as part of service provision; and (e) difficulties with method removal. Each of these are described below as they relate to community perceptions of service quality.

#### Interactions with providers

##### Initial reception from providers

Interactions between women and providers begin from the moment a woman arrives at the facility for FP care. Whether the woman experiences and/or expects positive or negative interactions with the facility staff, including the FP providers, is at the heart of perceived quality of family planning services. The nature of the initial interaction with the staff and provider was often a key aspect discussed during the FGD.

During the FGD, the majority of women across the different groups, whether discussing their own or others’ experiences, mentioned the initial interaction with the providers as crucial to forming perceptions of quality of care. This interaction, which is seen as the first contact with the provider, is essential for the construction of trust between health workers and clients of FP services. A good reception that includes being positively received was considered a sign of respect whereas a less warm welcome and poor treatment discouraged women/clients. Women perceive the mood of the provider at first contact as an important aspect of their satisfaction with the services they receive. A good welcome, according to the respondents, is generally reflected in the friendliness, the *"smile,"* the *"good words,"* and the warm or affectionate relationships that the providers show. For women, a good welcome gives confidence and reassures the woman and it allows her *to "open up"* and *"explain her real problems"* to the provider.

*"It's like what P3 said, if you go into a health center and the provider welcomes you with a smile, you'll be happy. So you can talk about your problems transparently to the health worker.”*

Bobo Dioulasso, unmarried women, users, ages 20–24

*"If a person welcomes you well, at the entrance even you understand it. He welcomed me with good humor, questioned me about my problems that I explained to him. Well, he didn't make a face at me. He spoke with a smile on his face."*

Banfora, married women, non-users, age 25–49

A positive reception and experience also encourages women to return to the clinic for follow-up services.

*"A provider did it in such a way that I wanted to return a second time. I was doing injectables and my menstruation had stopped. I went to explain to the provider who was on duty and he prescribed pills that made my period normal. The day he saw me again in consultation, he said, "Oh then my dear, what is it like? I say it's okay. He says, " oh great!" I was very happy with the way he welcomed me."*

Banfora, married women, users, age 25–49

On the other hand, a bad reception leads to negative perceptions and is often likely to discourage women from visiting providers for FP services and using FP methods.

*“I've never been but there's one of my girlfriends who says she's already been to the facility ward once and she found a lady, that the lady there received her coldly, that she had a drawn face and then she couldn't do what she went to do. She went another time and she found a man who received her well so she had the courage to say what she came for.”*

Banfora, unmarried women, non-users, age 20–24

Beyond the trust between service providers and clients that a good reception can create, it also reflects the respect and consideration that providers have for clients. This trust and respect encourage clients to return but also may lead to clients encouraging other women in their community to visit the FP clinic.

*“Everyone loves respect. When you take courage to go do something and you are respected, it motivates you more. But when you go to find that there are insults and that the person is mostly in a bad mood, you will feel his anger, it discourages you and you go home. So you will not be able to recommend the facility to someone else - on the contrary you will devalue it in the eyes of this person. This person may refuse to go.”*

Banfora, unmarried women, users, age 15–19

##### Quality of counseling

The majority of women in FGDs mentioned the importance of counseling, including the quality of information received during a consultation; this was often tied with a woman’s satisfaction with FP services. Many women mentioned that they view services as higher quality when the provider offers detailed information on each contraceptive method, including the benefits of each method, its potential side effects, and how to manage side effects. Notably, the women who talked about quality of counseling were typically the ones who had experience with method use.

For women, this detailed information is important because it helps to orient them on the choice of the most suitable method and prepares them to face the potential side effects and know how to deal with them.

*“There are some who say that the service from there is good that when you go there they take good care of you, they do not rush, they exchange with you so that you are happy with them, they show you how you should behave, how you should use the method, they show you the products and ask you to make a choice that will go with your needs, they show you everything, they tell you all this and then when you say what suits you and they offer you that”*

Banfora, married women, users, age 15–24

*"In my opinion, their job is good because they talk to us, they explain all the drugs to us. If you take that, here's what it causes. Or others even tell us that if you take this medicine and it causes you a problem, you can come back. All these were good manners."*

Bobo Dioulasso, married women, users, age 15–24.

For younger women and those who are single, these explanations are important insofar as they help them in the choice of their method, especially when it comes to their first use of contraception and the FP services.

*"When you go for the first time you are different from others because you have never done it. She [the provider] calls you and shows you all the methods and you make the choice of what you want, she will administer it to you or offer it to you. I liked it because someone can't make the choice for you. When you arrive there, they welcome you, those who have already been know what they have to do. But you who have never been, the health worker shows you all the methods. You choose what you want. They don't impose a method on you. I really liked this way of doing things. They show you all the methods and you choose. I like it because they let you choose.”*

Bobo Dioulasso, unmarried women, users, age 20–24

Also, high-quality counseling is likely to counter rumors and other misconceptions that are conveyed through social networks around contraceptive use and specific contraceptive methods and reassures women who may have had doubts and fears about use.

*"The idea is what they said there. They need to be made to understand the consequences. This makes most women who know about all these consequences be discouraged from family planning. And they will advise each other that the methods do not stand up, it is useless. They will use for two years, three years and have problems afterwards. So if they [providers] could explain to them what's next. That if you do this it will give certain advantages and that it helps you with this and that. And there they will integrate that. If that happens they would have been warned. It's much better"*

Bobo Dioulasso, unmarried women, non-users, age 20–24

##### Stigma and bias of providers

Respondents in some FGDs, especially those with young and single women, cited negative attitudes on the part of some providers. These include the judgment and stigma they show towards youth during consultations which young clients perceive to reflect problems with quality of care. These judgmental attitudes that were typically based on age and marital status include the view that young people should not be having early or premarital/non-marital sex. Such provider attitudes stand out as one of the aspects most negatively felt by young and unmarried women and are likely to discourage their visits to FP services. Thus, unmarried girls who are looking for contraceptive methods fear being stigmatized which serves as an important barrier to their accessing contraceptive services.

*“One of my aunts says she once went there when she was a young girl. When she arrived, they asked her, “You a little girl, you come to do the injection to do what?” They didn't respect her, I don't know how to say it... It upset her, she didn't like how she was treated."*

Bobo Dioulasso, unmarried women, non-users, age 20–24

*“What I want to add, she said that there are some when you go, they don't speak; it's as if they don't like to talk. There is a friend who told me that when she went, there was a female provider and she started insulting her; that she is a little girl, is she old enough to do methods? That she is small, that instead of working, it is to do methods and look for boys [sleeping with boys] that interests her. When my comrade told me this, I myself was afraid to go, they will tell me the same thing.*

Bobo Dioulasso, unmarried women, users, age 15–19

For respondents, the negative attitudes of providers may also relate to the choice of the method desired by young girls. The providers would not be in favor of young people asking for long-acting methods, in particular implants, and implicitly label them as having loose sexual morals.

*“What I do not like in their service, if you say that you come to put the norplant, the person [provider] welcomes you badly and tells you that you have not arrived anywhere [to say still a minor] and that it is the norplant that you want to put, that it is the boys that interest you, so when you leave, they reprimand you, that's what I don't like.”*

Bobo Dioulasso, unmarried women, users, age 15–19

*"They're not even going to go. My daughter went to a health center and the providers chased her away as a teenager. They refused to insert an implant into her. Because of that, she got pregnant. I gave her the money to go and do it and the providers sent her away. We can't say everything because if you want to talk too much it frustrates."*

Banfora, married women, non-users, age 25–49

##### Privacy and confidentiality

In the FGD with the younger and unmarried women, participants raised the importance of confidential FP service provision since contraceptive use remains taboo for this group. Meeting aunts, other family members, or other older community members in the same FP services causes discomfort for young and unmarried women. The breach of confidentiality during the search for care is therefore legitimately perceived by these women as a reason for dissatisfaction and a reason not to use the services.

*"No, this is not all due to the behavior of the providers but to the people in the waiting room for the FP service especially adults who are the age of our mothers. You, a young girl, if you go to sit there and if your neighbor or someone who is your mother's age finds you there and asks you what you came to do, often you will be forced to lie that you came to accompany someone and get up and disappear from the place now"*

Banfora, unmarried women, non-users, age 20–24

*“Which makes me not like to go to this place, because if you go there are benches like that, you sit down. I tell myself that if you go, people will look at you and say that hum it looks like these children love sex too much but because of norplant and others they do not get pregnant. So I calculate all this and it makes that I do not like to go to this place.”*

Banfora, unmarried women, users, age 15–19

The difficulty of maintaining confidentiality and privacy when visiting FP services by young people is linked to the way the facility is physically laid out in terms of organization of the reception areas and in relation to other services. In most health centers, respondents report that FP services are often provided in the same locations as, for example, maternal and neonatal care. In addition, there are also no separate spaces or specific schedules for young people.

*“I can say that there are barriers because it is a health center and there is only one public health center. So if you go you can meet one of your acquaintances and they can say that you have not reached a certain age and you come to do what here? Especially for us who are students, they will say that it is because you do not want to study that you come to do this. If you meet one of your aunts like this and you're going to have to turn around.”*

Banfora, unmarried women, users, age 15–19

Other women also deplored the fact that consultations may take place in the presence of several providers. Such a lack of privacy during FP consultations is also likely to cause discomfort and shame especially when it comes to methods to be inserted such as the intrauterine device (IUD).

*"Also my older brother's wife was going to get an IUD. When she went, in my opinion, the one who does the work there should just take her. The women should not be left so that others can come in and see her when she is naked. I didn't like that side of her experience. Whoever knows his work just has to go and do his job."*

Bobo Dioulasso, married women, non-users, age 25–49

#### Health system level factors

##### Wait time

Women also discussed the long wait times they usually experience at health centers. The older women who were in union were the main ones who mentioned this quality factor which is not surprising since they typically had more experience with FP services and may have had more household responsibilities awaiting their return. They felt the long wait time was related to the overload of work for providers and the fact that FP consultations and other care (e.g., prenatal and neonatal) take place in the same locations and are also offered by the same providers. Women felt that under these conditions, providers typically prioritized the maternal health visits over FP consultations. This causes long wait times or even postponement of FP appointments. Notably, a long wait time may affect use and FP visits, especially for those women who are trying to use a method covertly.

*“Often, when you go to the health center, you find that there are pregnant women they are weighing. You who came for FP they put you on hold. You will sit down and leave your fate in God's hand. They forget you, they will say that they will finish weighing pregnant women first before taking care of you. Yet, at home you hid to come and do it [FP]. Now, if you go to spend the day there, when you go back, nothing will be done, and there are other problems that you have created?”*

Bobo Dioulasso, married women, users, age 25–49

*" I'm going to the clinic [private clinic] now, because in the [private] clinic, if you get up at that time you arrive for FP, you can have some. But in public [clinic], if you leave, (chié!) you will sit waiting for a long time. Yet, people like us, we want to come back to cook. Now, if you have to get up go spend the whole morning [to get FP] that it's you who wants to do FP? You're not even going to get along with your mother-in-law, let alone with your husband. Now, if they could review that side.”*

Bobo Dioulasso, married women, users, age 25–49

Some of the younger, married women who had experience with family planning services also complained about problems with long wait times.

*“What we don't like sometimes you can happen to sit for a long time and you are told to come back after when you had already waited for a long time. You are told sometimes to come back tomorrow when you have already lost enough time, they let you sit down they come to pass you without saying anything now after a long wait they tell you to come back next time. You can last before someone comes to ask you what you want and you are told to come back after right now you see your time and essence in loss.”*

Banfora, married women, users, age 15–24

##### Stockouts of methods

During the focus groups, especially with older women and those who were married, the issue of the availability of contraceptive methods was raised. These experienced FP users noted dissatisfaction with the unavailability of contraceptive methods desired (i.e., stockouts) that forces women to use methods other than their preferred method. It also leads to wasted time finding the method either at the pharmacy or at another facility. This may lead to discontinuation or non-use of a method.

*“Most often if you arrive at the hospital, you find that the injection is stocked out, so you are forced to take the pill, then you come back to do the injection when it is available. Often there is not even the pill, you are forced to do what is available after you will come back to do what you want.”*

Bobo Dioulasso, married women, users, age 15–24

*"I have a friend who has already told me that she went there once to take a method and the midwife says that there was a stockout but she can leave and with what she had already been using she cannot get pregnant even in a month. She left and figuring that it was someone qualified who said it, she took this for real and she had sex and when she went again she was already pregnant, you see this kind of thing.”*

Banfora, unmarried women, non-users, age 20–24

##### Costs of services/methods

Since September of 2020, the Government of Burkina Faso made FP services, including contraceptive methods, free in public sector facilities [30]. During the FGD, some women described the non-implementation of this policy based on their experiences of going to facilities for a contraceptive method and being asked to pay for the method or the service. Further, women reported differential requests for payment based on familiarity with the provider; this was considered a sort of favoritism.

*"For me, because when you go for childbirth, they exchange well with you, and tell you that seven days after your delivery, you come back to do the FP and it's free. But when you go back to find another person, she asks you for a large sum. So, for me, there was a change at that level. That they are giving the same information even if there is a rotation of health workers that it is the same thing they are saying. That there is a change at this level.”*

Bobo Dioulasso, married women, users, age 25–49

*"What I want to ask doctors is to find unanimous prices for drugs. Or it is by relationship that they act. You can go to the hospital, they will do a free method for one person, the person will come and tell you. You are looking for some money to go, arrived they will give you a prescription you will go pay at pharmacy. If it's their relatives they do free, if they don't know you they take money.“*

Bobo Dioulasso, married women, users, age 15–24

##### Expectation for blood tests as part of service provision

In the FGDs, participants discussed their concerns about side effects of contraceptive methods. When asked about how these concerns about side effects related to service use and quality of services, women reported that higher quality of services were when providers offered blood tests to “verify the compatibility of methods with one’s body.” Indeed, according to a number of participants across the FGD with the different age groups and user statuses, to avoid the adverse effects of the methods it was felt that the providers, who have a better knowledge of the contraceptive methods, should carry out a battery of tests, including blood tests. These tests are considered important to determine which contraceptive method *"goes with the blood"* of each woman before deciding on the final choice of the method to be used. This, for them, will help reduce the side effects of contraceptive methods that many women experience and was related to being offered higher quality services.

*“I want that if you go [to the facility] that you are given a blood draw to do tests and see the method that suits your body before doing it [use FP] because you can go and make a choice of method and it will not suit your body but when you go to stop the method they tell you that it has not yet lasted long enough but if you do a blood sample for examinations before you use it there is no problem.”*

Banfora, married women, users, age 15–24

*"If they too could draw blood to see what suits you. As the big sister said if you go and it's norplant you want, that's what they give you. If it didn't suit you if you go back and you want the pills, you are told to go take the pills. But if they could give you what suits our blood it would please us.”*

Bobo Dioulasso, married women, users, age 25–49

*“For example, people don't have the same blood. The taking of the pills by some, leads to the change of date of the period. For others it is the implants that causes this. So if there was a method to check compatibility to avoid effects.”*

Banfora, unmarried women, non-users, age 15–19

Using blood tests to determine which method will best meet a client’s needs is not an established approach to FP service provision. Some methods do require checking blood pressure or potential risk factors prior to offering the method; however, there is no blood test that will tell a person what method is correct. This is discussed further in the discussion section.

##### Difficulties with method removal

In the focus groups, especially with the older women and those who are married, the problem with the withdrawal of long-acting contraceptive methods, particularly implants, was raised. These women discussed the difficulties encountered when they, or a friend, wanted to remove the implant following the experience of adverse effects of the method. In their view, the providers, for reasons that are not related to the client’s needs, refuse to withdraw the method or were unavailable to do so.

*“For me the most important thing, if you go to withdraw the method, they just have to be forgiving and accept to withdraw otherwise they make people work for it. Even when I went to remove my 2nd implant, they didn't want to do it. They made me jump through hoops a lot and I suffered.”*

Banfora, married women, non-users, age 25–49

*"Like what someone said here, if you insert the implant and go to remove it, the providers tell you heee there is no removal material. There is no time to withdraw, or it is these people who do it. You are forced to return home. Sometimes you come back, if they don't tell you that there is no anesthesia, they tell you that there is something else [so they cannot do it]. They have already done this to me even until I went to Mali to withdraw. When I arrived, the providers from Mali told me heee you have to come from Burkina Faso to do the withdrawal here? I said yes since I sought in vain in Burkina for the withdrawal and I did not get it. And then the implant had worsened my blood pressure, so I was told to remove it. There are difficulties regarding all this. They only have to forgive on this side. Remove! Or why refuse to withdraw?” (Speaking with an angry tone)*

Banfora, married women, non-users, age 25–49

*"True true there, if you go to put [the implant], it happens but if you go to remove it we are made to wait. We are even made to hang around. You can do three to four rounds in the hospital to get someone there, often if you have a relationship with someone at the hospital, he helps you remove it. I didn't like this experience."*

Bobo Dioulasso, married women, non-users, age 25–49

### Summary of results

Table 2 provides a summary of the results by age group, marital status, and contraceptive use experience. Overall, all women recognized the importance of interactions between providers and clients, including a welcoming and respectful visit and quality counseling. That said, some issues such as stigma and bias, and confidentiality and privacy were more salient for the youngest women and single women, who typically had less experience with facilities and methods and who were at greater risk of stigmatization due to premarital pregnancy (and sex) taboos. Indeed, for this category of women who use contraception in a context of social stigma because of their status, good relations with providers including a warm welcome and non-stigmatizing interactions are likely to build confidence and encourage future visits to a facility by the young women and their peers. In addition, a large proportion of the young people have little experience with maternal health and contraceptive services and methods. As a result, quality counseling is perceived by them as important for increasing their knowledge of contraceptive methods and to counter misinformation and other rumors related to their side effects.

**Table 2:** Quality characteristics identified as important by characteristics of FGD groups, Bobo Dioulasso and Banfora, Burkina Faso 2021

Group of women	Characteristics and experiences	Quality components important to all	Quality components important to specific groups of women
Young women Unmarried women	Under 25 years of age Unmarried No children No or little experience with FP Quality perceptions based on inputs from friends or family heard about at school, household, or community setting	Providers are friendly, welcoming, and respectful Counseling offered on the full range of methods Short wait time	Confidentiality and privacy offered/respected during visits Not stigmatizing questions and interactions with providers
Older women Married women	Ages 25 and older Married At least one child Current/prior FP experience Quality perceptions from their own experiences and from the experiences of others in their female network; discussions happen within family, at the market, or in other feminized spaces		Methods desired need to be available (no stock outs) Cost of methods and services Removal of implant available when desired Providers does a blood test to determine the best method for the woman

In terms of the health system-level factors, while most focus group discussions referred to issues like wait time, costs of methods and services, blood tests, and challenges with removal of long-acting methods, many of these aspects of service provision were specifically raised among the women who were older and in union, that is the women with more experience of method use. For these women, who are generally much less stigmatized in relation to FP use, the regular availability of contraceptive methods, waiting times and fear related to side effects are among the most important issues influencing their perceptions of service quality. Also, the possibility of withdrawal of contraceptive methods (long-term) at the right time and a belief that doing blood tests to help identify an appropriate method were important aspects raised by them.

## Discussion

This research aims to identify community perceptions of quality of care in FP services and differs from earlier work that typically examines quality from the client perspective [10–13, 15]. By identifying women at the community level and not immediately following a facility visit, this study obtained a broad array of perspectives on quality including from women with and without experience at facilities. This study showed that depending on women's characteristics, perceptions of quality are constructed either from women's personal experiences with methods and services or from stories told by other women. While older women and those in union tend to construct their discourses from their personal experiences, younger and single women structure theirs from the testimonies of others. The study also showed that women share their experiences (positive and negative) with other women in the community and, in turn, this sharing of experiences influences others’ perceptions of the quality of services of local facilities.

While overall some similar dimensions of service provision were mentioned by all women as criteria for assessing the quality of services, it was also noted that respondents differentially reported quality components by age, marital status and use experience. Younger women and those who are single are more socially stigmatized; these women emphasized the positive interactions with providers and confidentiality in the use of services as preferential criteria for quality assessment. Older women, on the other hand, focused on additional aspects of service delivery including the availability of methods, waiting times, and the difficulties getting implants removed. Notably, these same older women viewed quality services to mean that providers draw blood to help them “identify the method that goes best with her blood.” This sort of blood test is not available but this misperception that this equates to better service quality and less side effects is an important factor that needs to be addressed in future programs that seek to increase service use. Likewise, differential pricing or charging of clients was viewed as a frustrating aspect of service delivery.

Feeling welcomed and receiving in-depth counseling on a range of methods stand out as the aspects most mentioned by all women in their appreciation of the quality of FP services. A positive welcome is greatly appreciated, and a less warm welcome strongly criticized. On the other hand, counseling is typically mentioned when it is of higher quality and when women have appreciated it. Women who did not receive counseling did not generally report this as a bad experience with services. This may reflect that many women do not know what to expect during a FP visit and thus if they are not counseled, they do not necessarily know what they missed. Ensuring a positive welcome at the time of facility arrival and comprehensive counseling by providers can lead to improved community-level perceptions of service quality.

The components of quality important to women found here align well with what has been found previously in the literature through the seminal work by Bruce [9]. Women in the FGDs highlighted the importance of provision of full information and counseling on a range of methods by providers, particularly for the younger, less experienced women. As discussed above, when women were not counseled, this was not seen as poor quality since expectations of counseling were not necessarily the norm, especially among women who had not visited a facility. As found in other studies [10, 14, 15], provider competence and a welcoming facility environment were important areas identified across the FGDs as affecting perception of facility quality. This included the importance of providers being welcoming, friendly, and respectful. Likewise issues of stigma and discrimination by providers, previously shown in other studies [31, 32], were mentioned by the younger and unmarried FGD participants and were identified as important barriers to continued or future use of FP services. Availability of implant removal was raised by the more experienced FGD participants as an important quality concern reflecting multiple components of quality including lack of supplies, technical competence, and discrimination of providers who choose not to offer this service.

These results are also consistent with an earlier study among primary health care clients in Burkina Faso where important elements of perceived quality included health personnel practices and conduct and health care delivery [10]. The authors from the earlier Burkina Faso study identified gaps in the availability of commodities, the adequacy of the rooms and equipment, and costs of care as poorly reported quality elements [10]; these issues were important for perceived quality of FP services in our study as well in terms of stockouts, privacy, and inconsistent pricing. Finally, a qualitative study from Zambia demonstrated important health system factors that were related to perceived quality of FP services from the provider and community perspectives [24]. The factors identified as important for quality services in Zambia included provision of services by skilled providers, positive attitudes towards clients, and availability and affordability of methods [24, 25]. The findings from these earlier studies are consistent with the results found here for Burkina Faso that focuses specifically on the perceived quality of family planning services among a diverse set of women by age, marital status, and FP use experience.

It is also worth noting that respondents reported misperceptions around what quality of services entailed. In particular, women expected that providers will take blood and use that to recommend a correct method that “goes with one’s body.” When blood was not drawn, there was the feeling that the provider did not offer high quality services and clients may be dissatisfied with the services they received. This dissatisfaction, based on false expectations, may be relayed to friends and family and lead to low service utilization.

In the study context of two urban sites in Burkina Faso where facilities are over worked, understaffed, and under resourced, the lessons from this study and the WHO Global Standards for Quality health Care Services for Adolescents [33] can be useful for program strengthening. The WHO quality standards that relate to the FGD results include ensuring an appropriate package of services (e.g., information, counseling, treatment), provider competence (e.g., respect, privacy, confidentiality, non-discrimination, non-judgmental service provision), and facility characteristics (e.g., hours, welcoming, clean, supplies, etc.). While providers in study sties cannot be blamed for being tired and unavailable, they can be trained to be supportive to all clients, no matter the age, parity, and marital status. In addition, it may be pertinent to separate service days (or providers) for maternity health care (antenatal and postnatal care) and family planning services; this will reduce the burden on the providers and ideally reduce the wait time and provide additional privacy for clients. Further, programs seeking to improve the quality of services should also use job aids and posters that support the provision of comprehensive counseling that explains what providers can and cannot do as part of method provision, including that blood tests are not a standard of practice. In addition, it is important for outreach programs to address expectations for quality, including false expectations for blood testing. This may happen through the mass media that provides examples of a high-quality FP visit to help inform future clients’ expectations and demands for high quality counseling and services.

Before concluding, it is pertinent to discuss some of the limitations with this study. First, as this is a qualitative study, the results found here are not generalizable beyond the study cities and participants in the groups. This is particularly true since the two cities included in this study were places where the Beyond Bias program was working which may have affected the participants’ discussion, knowledge, and use of FP services. Second and relatedly, while there was an intervention that took place in some of the sites and not others, it was not possible to distinguish the responses by these program distinctions. This may relate to the small number of focus group discussions from each site or a lack of distinction in community-level perceived quality of services since all program activities took place at the facility level with no explicit community outreach. Third, rural areas were not included in this study to reduce the heterogeneity of the study sample. Thus, future studies on community perceptions of facility quality in rural Burkina Faso may be needed. Finally, women’s reports of quality perceptions may not include the full array of experiences and expectations if women were not comfortable talking in a group setting; thus, results are more normative than specific to the women’s experiences.

## Conclusions

This qualitative study provides recommendations for improving the quality of family planning services as well as the perceptions of quality based on specific elements that matter to women of different ages, marital statuses, and user experiences. Future programs should work with providers to ensure that they are offering services in a friendly and respectful manner. This goes beyond training of providers and also includes ensuring that providers are not overworked and discouraged with the tasks at hand. This may also require reorganizing facility-level services so that the same providers are not offering family planning and maternal and child health services in the same location at the same time; this will help to address some of the confidentiality and privacy concerns identified as well. Second, programs should undertake targeted communication and awareness activities either through the media and/or through posters or job aids at the facilities to address the incorrect belief about the necessity of blood tests equating to better quality services and the way to determine an appropriate method for a woman. This will also require training providers to offer complete information on a full range of methods during counseling sessions including the advantages and disadvantages of each. Further, providers need to be trained (and provided with relevant equipment) on removal of long-acting methods (implant and IUD) so that they are able to provide the full range of services needed in their facilities. In addition, ensuring that the policy of free provision of family planning methods and services is implemented in all public sector facilities will reduce concerns about favoritism and bias. Finally, ensuring that services are client-centered by providing quality services that take into account the women’s socio-cultural realities is crucial for supporting women’s adoption and continuation of family planning when they need or want it. It also supports their leaving the facility with a positive experience that they will share with others and may lead to their peers coming for services without hesitation when and if they need it. These approaches will help to meet the goal of universal access to family planning services.

## Data Availability

The datasets generated and/or analyzed during the current study are not publicly available in order to protect the identities of the participants involved but are available from the second author on reasonable request.

## Abbreviations

FGD: Focus group discussions
FP: Family planning
IDI: In-depth interview
ISSP: Institut Supérieur des Sciences de la Population
IUD: Intrauterine device
